# Convexity of Ruin Probability and Optimal Dividend Strategies for a General Lévy Process

**DOI:** 10.1155/2015/354129

**Published:** 2015-08-13

**Authors:** Chuancun Yin, Kam Chuen Yuen, Ying Shen

**Affiliations:** ^1^School of Mathematical Sciences, Qufu Normal University, Shandong 273165, China; ^2^Department of Statistics and Actuarial Science, The University of Hong Kong, Pokfulam Road, Hong Kong

## Abstract

We consider the optimal dividends problem for a company whose cash reserves follow a general Lévy process with certain positive jumps and arbitrary negative jumps. The objective is to find a policy which maximizes the expected discounted dividends until the time of ruin. Under appropriate conditions, we use some recent results in the theory of potential analysis of subordinators to obtain the convexity properties of probability of ruin. We present conditions under which the optimal dividend strategy, among all admissible ones, takes the form of a barrier strategy.

## 1. Introduction

In the literatures of actuarial science and finance, the optimal dividend problem is one of the key topics. For companies paying dividends to shareholders, a commonly encountered problem is to find a dividend strategy that maximizes the expected total discounted dividends until ruin. The pioneer work can be traced to de Finetti [1] who considered a discrete-time risk model with step sizes ±1 and showed that a certain barrier strategy maximizes the expected discounted dividend payments. Since then, the problem of finding the optimal dividend strategy has become a popular topic in the actuarial literature. For diffusion models, see, for example, Jeanblanc-Picqué and Shiryaev [2], Asmussen and Taksar [3], Gerber and Shiu [4], Løkka and Zervos [5], Paulsen [6], He and Liang [7], and Bai and Paulsen [8]. For the Cramér-Lundberg risk model, some related works on this subject include, among others, Gerber [9], Azcue and Muler [10, 11], Yuen et al. [12], Kulenko and Schmidli [13], Bai and Guo [14], and Hunting and Paulsen [15].

Analysis of optimal dividends for Lévy risk processes is of particular interest which have undergone an intensive development. For example, Avram et al. [16] considered a general spectrally negative Lévy process and gave a sufficient condition involving the generator of the Lévy process for the optimality of barrier strategy; Loeffen [17] showed that barrier strategy is optimal among all admissible strategies for general spectrally negative Lévy risk process with completely monotone jump density; Kyprianou et al. [18] relaxed this condition on the jump density; Yin and Wang [19] also studied the same problem and gave an alternate proof of the result; Loeffen [20, 21] considered the optimal dividend problem with transaction costs and a terminal value for the spectrally negative Lévy process. Recently, Bayraktar et al. [22] using the fluctuation theory of spectrally positive Lévy processes show the optimality of barrier strategies for all such Lévy processes. See Yin and Wen [23] for a different approach. All of the above mentioned works are based on spectrally one-sided models. There are, however, few papers that studied the analogous problems for Lévy process with two-sided jumps (cf. Bo et al. [24, 25]). Inspired by the works of Avram et al. [16], Loeffen [17], and Kyprianou et al. [18], Yuen and Yin [26] considered the optimal dividend problem for a special Lévy process with both upward and downward jumps and showed that the optimal strategy takes the form of a barrier strategy if the Lévy measure (both negative and positive jumps) has a completely monotone density. The purpose of the present paper is to extend the result of Yuen and Yin [26] to the case with less restrictive conditions on the Lévy measure. Although the broader case definitely makes the optimization problem more challenging and complex, recent results on the theory of potential analysis of subordinators can be applied to handle it. In particular, our main results show that the optimal dividend strategy is still of a barrier type if the Lévy process has certain positive jumps and Lévy density of negative jumps is completely monotone or log-convex.

The paper is organized as follows. In Section 2, we introduce the mathematical formulation of the problem. In Section 3, we give a brief review on ladder processes and potential measure for general Lévy processes. The convexities of the ruin probability and the scale function are discussed in Sections 4 and 5 and the main results and their proofs are given in Section 6.

## 2. The Model

To present the mathematical formulation of the problem of study, let us first introduce some notations and definitions. Let *X* = {*X*_*t*_}_*t*≥0_ be a real-valued Lévy process on a filtered probability space (*Ω*, *ℱ*, *𝔽*, *𝒫*) where *𝔽* = (*ℱ*_*t*_)_*t*≥0_ is generated by the process *X* and satisfies the usual conditions of right continuity and completeness. Denote by *P*_*x*_ the law of *X* when *X*_0_ = *x*. Let *E*_*x*_ be the expectation associated with *P*_*x*_. For notational convenience, we write *P* and *E* when *X*_0_ = 0. Write the Lévy triplet of *X* as (*a*, *σ* ^2^, Π), where *a*, *σ* ≥ 0 are real constants and Π is a positive measure on (−*∞*, *∞*)∖{0} which satisfies the integrability condition

(1) $$\begin{array}{c} \overset{\infty }{\underset{-\infty }{\int }}\left(1\wedge x^{2}\right)\Pi \left(dx\right)<\infty . \end{array}$$

If Π(*dx*) = *π*(*x*)*dx*, then we call *π* the Lévy density. The characteristic exponent of *X* is given by

(2) κθ=−1tlogEeiθXt=−iaθ+12σ2θ2+∫−∞∞1−eiθx+iθx1|x|<1Πdx,

where 1_*A*_ is the indicator of set *A*. Furthermore, define the Laplace exponent of *X* by

(3) Ψθ=1tlogEeθXt= aθ+12σ2θ2+∫−∞∞eθx−1−θx1|x|<1Πdx.

Such a Lévy process is of bounded variation if and only if *σ* = 0 and ∫_−1_^1^ | *x* | Π(*dx*) < *∞*. If Π{(0, *∞*)} = 0, then the Lévy process *X* with no positive jumps is called the spectrally negative Lévy process; if Π{(−*∞*, 0)} = 0, then the Lévy process *X* with no negative jumps is called the spectrally positive Lévy process. It is usual to assume that *P*(lim_*t*→*∞*_ *X*_*t*_ = +*∞*) = 1 which says nothing other than Ψ′(0+) > 0. For more information on Lévy processes we refer to the excellent book by Kyprianou [27].

Now, we consider an insurance company or investment company whose cash reserve process (also called risk process or surplus process) evolves according to the process *X* before dividends are deducted. Let *ξ* = {*L*_*t*_^*ξ*^ : *t* ≥ 0} be a dividend policy consisting of a right-continuous nonnegative nondecreasing process adapted to the filtration {*ℱ*_*t*_}_*t*≥0_ of *X* with *L*_0−_^*ξ*^ = 0, where *L*_*t*_^*ξ*^ represents the cumulative dividends paid up to time *t*. Given a control policy *ξ*, the controlled reserve process with initial capital *x* ≥ 0 is given by *U* ^*ξ*^ = {*U*_*t*_^*ξ*^ : *t* ≥ 0} where

(4) $$\begin{array}{c} U_{t}^{\xi }=X_{t}-L_{t}^{\xi }, \end{array}$$

with *X*_0_ = *x*. Let *τ* ^*ξ*^ = {*t* > 0 : *U*_*t*_^*ξ*^ < 0} be the ruin time when dividend payments are taken into account. Define the value function associated to dividend policy *ξ* by

(5) $$\begin{array}{c} V_{\xi }\left(x\right)=E_{x}\left(\overset{\tau^{\xi }}{\underset{0}{\int }}e^{-\delta t}dL_{t}^{\xi }\right), \end{array}$$

where *δ* > 0 is the discounted rate. The integral is understood pathwise in a Lebesgue-Stieltjes sense. Clearly, *V*_*ξ*_(*x*) = 0 for *x* < 0. A dividend policy is called admissible if *L*_*t*_^*ξ*^ − *L*_*t*−_^*ξ*^ ≤ *U*_*t*_^*ξ*^ for *t* < *τ* ^*ξ*^ and *L*_*τ*^*ξ*^_^*ξ*^ − *L*_*τ*^*ξ*^−_^*ξ*^ = 0 for *τ* ^*ξ*^ < *∞*. Denote by Ξ the set of all admissible dividend policies. Our objective is to find

(6) $$\begin{array}{c} V_{*}\left(x\right)=\underset{\xi \in \Xi }{sup}V_{\xi }\left(x\right), \end{array}$$

and an optimal policy *ξ* ^*∗*^ ∈ Ξ such that *V*_*ξ*^*∗*^_(*x*) = *V*_*∗*_(*x*) for all *x* ≥ 0. The function *V*_*∗*_ is called the optimal value function.

We denote by *ξ*_*b*_ = {*L*_*t*_^*b*^ : *t* ≥ 0} the barrier strategy at *b* and let *U* ^*b*^ be the corresponding risk process; that is, *U*_*t*_^*b*^ = *X*_*t*_ − *L*_*t*_^*b*^. Note that *ξ*_*b*_ ∈ Ξ. Also, if *U*_0_^*b*^ ∈ [0, *b*], then the process *L*_*t*_^*b*^ can be explicitly represented by

(7) $$\begin{array}{c} L_{t}^{b}=\left(\underset{s\leq t}{sup}X_{s}-b\right)\vee 0. \end{array}$$

If *U*_0_^*b*^ = *x* > *b*, then

(8) $$\begin{array}{c} L_{t}^{b}=\left(x-b\right)1_{\left\{t=0\right\}}+\left(\underset{s\leq t}{sup}X_{s}-b\right)\vee 0. \end{array}$$

Denote by *V*_*b*_(*x*) the dividend value function if barrier strategy *ξ*_*b*_ is applied; that is,

(9) $$\begin{array}{c} V_{b}\left(x\right)=E_{x}\left(\overset{\tau^{\xi_{b}}}{\underset{0}{\int }}e^{-\delta t}dL_{t}^{b}\right). \end{array}$$

Applying Ito's formula for semimartingale, we can prove that *V*_*b*_ is the solution to

(10) $$\begin{array}{c} \Gamma V_{b}\left(x\right)=\delta V_{b}\left(x\right),\;x>0,\\ V_{b}\left(x\right)=0,\;x<0,\\ V_{b}\left(0\right)=0,\;\sigma^{2}>0,\\ V_{b}^{\prime }\left(b\right)=1,\\ V_{b}\left(x\right)=x-b+V_{b}\left(b\right), \end{array}$$

where Γ is the infinitesimal generator of *X* with

(11) Γgx=12σ2g′′x+ag′x+∫−∞∞gx+y−gx−g′xy1y<1   ×Πdy.

In the sequel, we assume that, for any *δ* > 0, the equation Ψ(*z*) = *δ* has a unique solution on (0, *∞*), say *ρ*(*δ*). A typical example is that the Lévy measure of the positive jumps has the following gamma distribution Γ(*r*, 1/*γ*); that is,

(12) $$\begin{array}{c} P\left(x\right)=\overset{x}{\underset{0}{\int }}\frac{r^{1/\gamma }}{\Gamma \left(1/\gamma \right)}y^{1/\gamma -1}e^{-ry}dy,\;x>0, \end{array}$$

where *r* is a positive number and *γ* is an even number.

Following similar reasoning to Yuen and Yin [26], *V*_*b*_ can be expressed as

(13) $$\begin{array}{c} V_{b}\left(x\right)=\left\{\begin{array}{cc} \frac{h\left(x\right)}{h^{\prime }\left(b\right)}, & 0\leq x\leq b,\\ x-b+\frac{h\left(b\right)}{h^{\prime }\left(b\right)}, & x>b, \end{array}\right. \end{array}$$

where

(14) $$\begin{array}{c} h\left(x\right)=\left[1-\tilde{\psi }\left(x\right)\right]e^{\rho \left(\delta \right)x}. \end{array}$$

Here, let $\tilde{\psi }(u)$ be the ruin probability for a Lévy process $\tilde{X}$ with Laplace exponent *ψ*_*ρ*(*δ*)_ given by *ψ*_*ρ*(*δ*)_(*η*) = Ψ(*η* + *ρ*(*δ*)) − *δ*. Note that the process $\tilde{X}$ has the Lévy triplet $\left(\tilde{a},{\tilde{\sigma }}^{2},\tilde{\Pi }\right)$, where ${\tilde{\sigma }}^{2}=\sigma^{2}$, $\tilde{\Pi }(dx)=e^{\rho (\delta )x}\Pi (dx)$, and

(15) $$\begin{array}{c} \tilde{a}=a+\sigma^{2}\rho \left(\delta \right)+\overset{k}{\underset{-\infty }{\int }}\left(e^{\rho \left(\delta \right)y}-1\right)y1_{\left\{|y|\leq 1\right\}}\Pi \left(dy\right). \end{array}$$

Moreover,

(16) $$\begin{array}{c} \int_{|x|\geq 1}e^{\rho \left(\delta \right)x}\Pi \left(dx\right)<\infty . \end{array}$$

## 3. Some Results on Ladder Processes and Potential Measure

In this section, we recap some basic facts about ladder processes and potential measure. Consider the dual process *Y* = {*Y*_*t*_}_*t*≥0_, with *Y*_0_ = 0, where *Y*_*t*_ = −*X*_*t*_, *t* ≥ 0. It is easy to see that the Lévy triplet of *Y* is (−*a*, *σ* ^2^, Π_*Y*_), where Π_*Y*_(*dx*) = *π*_*X*_(−*x*)*dx*. Let

(17) $$\begin{array}{c} \underline{Y_{t}}=\underset{0\leq s\leq t}{inf}Y_{s},\;\;{\bar{Y}}_{t}=\underset{0\leq s\leq t}{sup}Y_{s} \end{array}$$

be the processes of the first infimum and the last supremum of the Lévy process *Y*, respectively. Following Klüppelberg et al. [28], we now introduce the notion of ladder processes and potential measure. Let *L* = {*L*_*t*_ : *t* ≥ 0} denote the local time in the time period [0, *t*] that $\bar{Y}-Y$ spends at zero. Then *L* ^−1^ = {*L*_*t*_^−1^ : *t* ≥ 0} is the inverse local time such that *L*_*t*_^−1^ = inf{*s* ≥ 0 : *L*_*s*_ > *t*}, where we take the infimum of the empty set as *∞*. Define an increasing process *H* by {*H*_*t*_ = *Y*_*L*_*t*_^−1^_ : *t* ≥ 0}, that is, the process of new maxima indexed by local time at the maximum. The processes *L* ^−1^ and *H* are both defective subordinators, and we call them the ascending ladder time and ladder height process of *Y*, respectively. It is understood that *H*_*t*_ = *∞* when *L*_*t*_^−1^ = *∞*. Throughout the paper, we denote the nondefective versions of *L*, *L* ^−1^, and *H* by *ℒ*, *ℒ* ^−1^, and *ℋ*, respectively. In fact, the pair (*ℒ* ^−1^, *ℋ*) is a bivariate subordinator. Define $\left({\hat{L}}^{-1},\hat{H}\right)$ the descending ladder time and the ladder height processes in an analogous way. Note that $\hat{H}$ is a process which is negatively valued. Because *Y* drifts to −*∞*, the decreasing ladder height process is not defective. Associated with the ascending and descending ladder processes are the bivariate renewal functions *U* and $\hat{U}$. The former is defined by

(18) $$\begin{array}{c} U\left(dx,ds\right)=\overset{\infty }{\underset{0}{\int }}P\left(H_{t}\in dx,L_{t}^{-1}\in ds\right)dt. \end{array}$$

Taking Laplace transforms shows that

(19) $$\begin{array}{c} \overset{\infty }{\underset{0}{\iint }}e^{-\beta x-\alpha s}U\left(dx,ds\right)=\frac{1}{k\left(\alpha ,\beta \right)},\;\alpha ,\beta \geq 0, \end{array}$$

where *k*(*α*, *β*) is its joint Laplace exponent such that

(20) $$\begin{array}{c} k\left(0,\beta \right)=q+c\beta +\int_{\left(0,\infty \right)}\left(1-e^{-\beta x}\right)\Pi_{H}\left(dx\right), \end{array}$$

*q* ≥ 0 is the killing rate of *H* so that *q* > 0 if and only if lim_*t*→*∞*_ *Y*_*t*_ = −*∞*, *c* ≥ 0 is the drift of *H*, and Π_*H*_ is its jump measure. Denote the marginal measure of *U*(·, ·) by

(21) Udx=Udx,0,∞=∫0∞PHt∈dxdt=∫0∞e−qtPHt∈dxdt, x≥0.

The function *U* is called the potential/renewal measure. As for the descending ladder process, $\hat{U}$ and $\hat{k}$ are defined similarly. Write Π_+_ and Π_−_ for the restrictions of Π(*du*) and Π(−*du*) to (0, *∞*). Furthermore, for *u* > 0, define

(22) $$\begin{array}{c} {\bar{\Pi }}_{Y}^{+}\left(u\right)=\Pi_{Y}\left\{\left(u,\infty \right)\right\},\\ {\bar{\Pi }}_{Y}^{-}\left(u\right)=\Pi_{Y}\left\{\left(-\infty ,-u\right)\right\},\\ {\bar{\Pi }}_{Y}\left(u\right)={\bar{\Pi }}_{Y}^{+}\left(u\right)+{\bar{\Pi }}_{Y}^{-}\left(u\right). \end{array}$$

We next introduce the notions of a special Bernstein function and complete Bernstein function and two useful results. Recall that a function *ϕ* : (0, *∞*)→(0, *∞*) is called a Bernstein function if it admits a representation

(23) $$\begin{array}{c} \phi \left(\lambda \right)=a+b\lambda +\overset{\infty }{\underset{0}{\int }}\left(1-e^{-\lambda x}\right)\mu \left(dx\right), \end{array}$$

where *a* ≥ 0 is the killing term, *b* ≥ 0 is the drift, and *μ* is the Lévy measure concentrated on (0, *∞*) satisfying ∫_0_^*∞*^(1∧*x*)*μ*(*dx*) < *∞*. A function *ψ* is called a special Bernstein function if the function *ψ*(*λ*) = *λ*/*ϕ*(*λ*) is again a Bernstein function. Let

(24) $$\begin{array}{c} \psi \left(\lambda \right)=\tilde{a}+\tilde{b}\lambda +\overset{\infty }{\underset{0}{\int }}\left(1-e^{-\lambda x}\right)\nu \left(dx\right) \end{array}$$

be the corresponding representation. It was shown in Song and Vondraček [29] that

(25) $$\begin{array}{c} \tilde{b}=\frac{1}{a+\mu \left(\left(0,\infty \right)\right)}1_{\left\{b=0\right\}},\;\\ \tilde{a}=\frac{1}{b+\int_{0}^{\infty }t\mu \left(dt\right)}1_{\left\{a=0,\mu \left(0,\infty \right)<\infty \right\}}. \end{array}$$

A possibly killed subordinator is called a special subordinator if its Laplace exponent is a special Bernstein function. Song and Vondraček [30] showed that a sufficient condition for *ϕ* to be a special subordinator is that *μ*(*x*, *∞*) is log-convex on (0, *∞*). A function *ϕ* : (0, *∞*) → *ℝ* is called a complete Bernstein function if there exists a Bernstein function *η* such that

(26) $$\begin{array}{c} \phi \left(\lambda \right)=\lambda^{2}L\eta \left(\lambda \right),\;\lambda >0, \end{array}$$

where *ℒ* stands for the Laplace transform. It is known that every complete Bernstein function is a Bernstein function and that the following three conditions are equivalent:

(i) *ϕ*  is a complete Bernstein function;
(ii) *ψ*(*λ*) = *λ*/*ϕ*(*λ*) is a complete Bernstein function;
(iii) *ϕ* is a Bernstein function whose Lévy measure *μ* is given by
  (27) $$\begin{array}{c} \mu \left(dt\right)=dt\overset{\infty }{\underset{0}{\int }}e^{-st}\nu \left(ds\right), \end{array}$$

where *ν* is a measure on (0, *∞*) satisfying

(28) $$\begin{array}{c} \overset{1}{\underset{0}{\int }}\frac{1}{s}\nu \left(ds\right)<\infty ,\;\;\overset{\infty }{\underset{1}{\int }}\frac{1}{s^{2}}\nu \left(ds\right)<\infty . \end{array}$$

To end the section, we present two results which are useful in potential theory and will be used in later sections of the paper. The first due to Kyprianou et al. [18] (see also Song and Vondraček [30]) is summarized in Lemma 1 while the second due to Kingman [31] and Hawkes [32] is given in Lemma 2.

**Lemma 1 .**

Let *H* be a subordinator whose Lévy density, say *μ*(*x*), *x* > 0, is log-convex. Then, the restriction of its potential measure to (0, *∞*) has a nonincreasing and convex density. Furthermore, if the drift of *H* is strictly positive, then the density is in *C* ^1^(0, *∞*).

**Lemma 2 .**

Suppose that *H* is a subordinator with Laplace exponent *ϕ* and potential measure *U*. Then, *U* has a density *u* which is completely monotone on (0, *∞*) if and only if the tail of the Lévy measure is completely monotone.

**Remark 3 .**

Note that the tail of the Lévy measure *μ* is a completely monotone function if and only if *μ* has a completely monotone density. Thus, we have the following two equivalent statements: *ϕ* is a complete Bernstein function if and only if *U* has a density *u* which is completely monotone on (0, *∞*); or, equivalently, *U* has a density *u* which is completely monotone on (0, *∞*) if and only if *μ* has a completely monotone density.

## 4. Convexity of Probability of Ruin

Define the probability of ruin by

(29) ψx=Pthere  exists  t≥0  such  that  x+Xt≤0=Pthere  exists  t≥0  such  that  Yt≥x.

It follows from Bertoin and Doney [33] that *ψ*(*x*) = *αU*(*x*, *∞*), where *α* ^−1^ = *U*(0, *∞*) = ∫_0_^*∞*^ *P*(*H*_*t*_ < *∞*)*dt*, with *U* given in (21).

For simplicity, we write the Lévy measure Π as

(30) $$\begin{array}{c} \Pi \left(dx\right)=\left\{\begin{array}{cc} \Pi_{+}\left(dx\right), & x>0,\\ \pi_{-}\left(-x\right)dx, & x<0. \end{array}\right. \end{array}$$

Recall that an infinitely differentiable function *f* ∈ (0, *∞*)→[0, *∞*) is called completely monotone if (−1)^*n*^ *f* ^(*n*)^(*x*) ≥ 0 for all *n* = 0,1, 2,… and all *x* > 0.

**Lemma 4 (see Vigon [34]).**

For the Lévy process *X*, one has

(31) Π¯Hx=−∫−∞0U^dyΠ¯Y+x−y=−∫−∞0U^dyΠ¯X  −x−y, x>0,

where *Y* = −*X* and $\hat{U}$ is the potential measure corresponding to $\hat{H}$.

**Theorem 5 .**

(i) Suppose *π*_−_ is completely monotone on (0, *∞*). Then, the probability of ruin *ψ* is completely monotone on (0, *∞*). In particular, *ψ* ∈ *C* ^*∞*^(0, *∞*).

(ii) Suppose *π*_−_ is log-convex on (0, *∞*). Then,

(a) *ψ* is convex on (0, *∞*);
(b) *ψ*′ is concave on (0, *∞*);
(c) if *X* has no Gaussian component, then *ψ* is twice continuously differentiable except at finitely or countably many points on (0, *∞*), else *ψ* ∈ *C* ^2^(0, *∞*).

**Proof**

We first prove (i). Since *π*_−_ is completely monotone on (0, *∞*), it follows from Lemma 4 that the tail Π_*ℋ*_(*x*, *∞*) of Lévy measure Π_*ℋ*_ is a complete monotone function. Also, it follows from Lemma 2 that the potential measure *U* has a density *u* which is completely monotone on (0, *∞*). Thus, the probability of ruin *ψ* is completely monotone on (0, *∞*) as *ψ*(*x*) = *αU*(*x*, *∞*).

We now prove (ii). The log-convexity of *π*_−_ implies the log-convexity of ${\bar{\Pi }}_{Y}^{+}$, and hence ${\bar{\Pi }}_{ℋ}$ is log-convex on (0, *∞*) due to Lemma 4 as log-convexity is preserved under mixing. It follows from Lemma 1 that the potential measure *U* has a nonincreasing and convex density *u*. Thus, *ψ*′ = −*αu* is nondecreasing and concave on (0, *∞*), and hence (a) and (b) are proved. Since a convex function on (0, *∞*) is differentiable except at finitely or countably many points, we see that *ψ* is twice continuously differentiable except at finitely or countably many points on (0, *∞*) if *X* has no Gaussian component. On the other hand, if *X* has a Gaussian component or, equivalently, the drift of ascending ladder processes *H* strictly positive, then it follows from Lemma 1 that *u* ∈ *C* ^1^(0, *∞*), and hence *ψ* ∈ *C* ^2^(0, *∞*). Therefore, (c) is proved.

## 5. Convexity of *h*

For *h* in (14), define a barrier level by

(32) $$\begin{array}{c} b^{*}=\sup\left\{b\geq 0:h^{\prime }\left(b\right)\leq h^{\prime }\left(x\right)\;\forall x\geq 0\right\}, \end{array}$$

where *h*′(0) is understood to be the right-hand derivative at 0.

For a spectrally negative Lévy process, that is, in the case of Π{(0, *∞*)} = 0, it was shown in Loeffen [17] that the derivative of the *δ*-scale function *W* ^(*δ*)′^(*x*) is convex for *δ* > 0 if Π(*x*, *∞*) is completely monotone. This implies that there exists an *a* ^*∗*^ ≥ 0 such that *W* ^(*δ*)^ is concave on (0, *a* ^*∗*^) and convex on (*a* ^*∗*^, *∞*). Also, Kyprianou et al. [18] showed that if Π(*x*, *∞*) has a density on (0, *∞*) which is nonincreasing and log-convex; then, for each *δ* ≥ 0, the scale function *W* ^(*δ*)^(*x*) and its first derivative are convex beyond some finite value of *x*.

Parallel to the results of Loeffen [17] and Kyprianou et al. [18] for spectrally negative Lévy processes, we have the following results.

**Theorem 6 .**

(i) Suppose *π*_−_ is completely monotone on (0, *∞*). Then, the derivative *h*′(*u*) is strictly convex on (0, *∞*) and *h* ∈ *C* ^*∞*^(0, *∞*).

(ii) Suppose *π*_−_ is log-convex on (0, *∞*). Then, the *h* and its derivative *h*′ are strictly convex on (*b* ^*∗*^, *∞*). Moreover, if *X* has no Gaussian component, *h* is twice continuously differentiable except at finitely or countably many points on (0, *∞*), else *h* ∈ *C* ^2^(0, *∞*).

**Proof**

Since *π*_−_ is completely monotone on (0, *∞*), we have ${\tilde{\pi }}_{-}$ which is also completely monotone on (0, *∞*), where $\tilde{\Pi }(dx)={\tilde{\pi }}_{-}(-x)dx$,  *x* < 0. We can now apply Theorem 5 to deduce that the probability of ruin $\tilde{\psi }$ is completely monotone on (0, *∞*). In particular, $\tilde{\psi }\in C^{\infty }(0,\infty )$. It is easy to prove that *h*′(*u*) is strictly convex on (0, *∞*) and *h* ∈ *C* ^*∞*^(0, *∞*). Hence, (i) is proved.

Let $\tilde{ℋ}$ ($\hat{\tilde{ℋ}}$) be the ascending (descending) ladder height process of $\tilde{Y}=-\tilde{X}$. By Lemma 4, we have

(33) $$\begin{array}{c} {\bar{\Pi }}_{\tilde{H}}\left(x\right)=-\overset{0}{\underset{-\infty }{\int }}\hat{\tilde{U}}\left(dy\right){\bar{\Pi }}_{\tilde{X}}^{-}\left(x-y\right),\;x>0, \end{array}$$

where $\hat{\tilde{U}}$ is the renewal measure corresponding to $\hat{\tilde{H}}$. Then,

(34) ΠH~′x=−eρδx∫−∞0U~^dye−ρδyπ−y−x≡ eρδxν+x.

The assumption of log-convexity of *π*_−_ implies that *ν*_+_ is log-convex, and hence $\Pi_{\tilde{ℋ}}^{\prime }(x)$ is also log-convex. It follows from Lemma 1 of Kyprianou and Rivero [35] that the restriction of its potential measure to (0, *∞*) of a subordinator with Lévy density *ν*_+_ has a nonincreasing and convex density, say *f*_*δ*_. Also, the restriction of its potential measure to (0, *∞*) of a subordinator with Lévy density $\Pi_{\tilde{ℋ}}^{\prime }(x)$ has a nonincreasing and convex density, say *h*_*δ*_. Moreover, *h*_*δ*_(*x*) = *e* ^*ρ*(*δ*)*x*^ *f*_*δ*_(*x*). Thus, ${\tilde{\psi }}^{\prime }(x)=-\tilde{\alpha }e^{\rho (\delta )x}f_{\delta }(x)$, where ${\tilde{\alpha }}^{-1}=\int_{0}^{\infty }P({\tilde{H}}_{t}<\infty )dt$. Since $h(x)=[1-\tilde{\psi }(x)]e^{\rho (\delta )x}$, we have

(35) $$\begin{array}{c} h^{\prime }\left(x\right)=\rho \left(\delta \right)h\left(x\right)+\alpha f_{\delta }\left(x\right),\;x>0. \end{array}$$

This implies that *h*′(*x*) tends to *∞* as *x* tends to *∞* as lim_*x*→*∞*_ *h*(*x*) = *∞*. Thus *b* ^*∗*^ < *∞*. Applying the same arguments as those in Kyprianou et al. [18], we can prove that *h* and its derivative *h*′ are strictly convex on (*b* ^*∗*^, *∞*). Finally, the smoothness of *h* is a direct consequence of Theorem 5. So, (ii) is proved.

## 6. Main Results and Proofs

We now present the main results of the paper about the optimality of the barrier strategy *ξ* ^*b*^*∗*^^ for de Finetti's dividend problem for general Lévy processes. This is a continuation of the work of Yuen and Yin [26] in which a special Lévy process with both upward and downward jumps and a completely monotone density was considered.

**Theorem 7 .**

Suppose that *ν* is a nonnegative function on (0, *∞*) which is sufficiently smooth and satisfies the following:

(i) (Γ − *δ*)*ν*(*x*) ≤ 0, for almost every *x* > 0;
(ii) *ν* is concave on (0, *∞*);
(iii) *ν*′(*x*) ≥ 1, *x* > 0.

Then, *ν*(*x*) ≥ *V*_*∗*_(*x*).

**Theorem 8 .**

Suppose that *V*_*b*_ defined in (13) is sufficiently smooth and satisfies

(i) *V*_*b*_′(*x*) > 1, for all *x* ∈ [0, *b*;
(ii) (Γ − *δ*)*V*_*b*_(*x*) ≤ 0, for all  *x* > *b*.

Then, *V*_*b*_(*x*) = *V*_*∗*_(*x*). In particular, if (Γ − *δ*)*V*_*b*^*∗*^_(*x*) ≤ 0, for all *x* > *b* ^*∗*^, then *V*_*b*^*∗*^_(*x*) = *V*_*∗*_(*x*).

**Theorem 9 .**

Suppose that *π*_−_ is completely monotone. Then, *V*_*b*^*∗*^_(*x*) = *V*_*∗*_(*x*); that is, the barrier strategy at *b* ^*∗*^ is the optimal strategy among all admissible strategies.

**Theorem 10 .**

Suppose that *π*_−_ is log-convex on (0, *∞*). Then, *V*_*b*^*∗*^_(*x*) = *V*_*∗*_(*x*); that is, the barrier strategy at *b* ^*∗*^ is the optimal strategy among all admissible strategies.

Before proving the main results, we give two lemmas which are similar to those in Loeffen [17] for spectrally negative Lévy processes.

**Lemma 11 .**

Suppose that *h* is sufficiently smooth and convex in the interval (*b* ^*∗*^, *∞*). Then, the following statements hold:

(i) *b* ^*∗*^ < *∞*;
(ii) *V*_*b*^*∗*^_′(*x*) ≥ 1 for *x* ∈ [0, *b* ^*∗*^] and *V*_*b*^*∗*^_′(*x*) = *V*_*x*_′(*x*) = 1 for *x* > *b* ^*∗*^;
(iii) (Γ − *δ*)*V*_*b*^*∗*^_(*x*) = 0 for *x* ∈ (0, *b* ^*∗*^).

**Proof**

As lim_*x*→*∞*_ *h*′(*x*) = *∞*, we have (i). For (ii), *V*_*b*^*∗*^_′(*x*) = *h*′(*x*)/*h*′(*b* ^*∗*^) for *x* ∈ [0, *b* ^*∗*^]; it follows from the definition of *b* ^*∗*^ that *V*_*b*^*∗*^_(*x*) ≥ 1 for *x* ∈ [0, *b* ^*∗*^]; *V*_*b*^*∗*^_′(*x*) = *V*_*x*_′(*x*) = 1 for *x* > *b* ^*∗*^ because of *V*_*b*^*∗*^_(*x*) = *x* − *b* ^*∗*^ + *V*_*b*^*∗*^_(*b* ^*∗*^); and *V*_*x*_′(*x*) = 1 since *V*_*x*_(*x*) = *h*(*x*)/*h*′(*x*). Finally, (iii) is due to (Γ − *δ*)*h*(*x*) = 0 for *x* ∈ (0, *b* ^*∗*^) and (13).

**Lemma 12 .**

Suppose that *h* is sufficiently smooth and is convex in the interval (*b* ^*∗*^, *∞*). Then, for *x* > *b* ^*∗*^,

(i) *V*_*b*^*∗*^_′′(*x*) = 0 ≤ *V*_*x*_′′(*x*−) if *σ* ≠ 0;
(ii) *V*_*b*^*∗*^_′(*y*) ≥ *V*_*x*_′(*y*), *y* ∈ [0, *x*];
(iii) *V*_*b*^*∗*^_(*x*) ≥ *V*_*x*_(*x*);
(iv) (Γ − *δ*)*V*_*b*^*∗*^_(*x*) ≤ 0.

**Proof**

If *σ* ≠ 0, *V*_*b*^*∗*^_′′(*x*) = 0 is clear. Also, since *h* ∈ *C* ^2^(0, *∞*) and is convex in the interval (*b* ^*∗*^, *∞*), we have *V*_*x*_′′(*x*−) = lim_*y*↑*x*_ *V*_*x*_′′(*y*) = lim_*y*↑*x*_ *h*′′(*y*)/*h*′(*x*) ≥ 0. Thus, (i) is proved.

For *y* ∈ [0, *b* ^*∗*^], by the definition of *b* ^*∗*^, we have

(36) $$\begin{array}{c} V_{b^{*}}^{\prime }\left(y\right)-V_{x}^{\prime }\left(y\right)=\frac{h^{\prime }\left(y\right)}{h^{\prime }\left(b^{*}\right)}-\frac{h^{\prime }\left(y\right)}{h^{\prime }\left(x\right)}\geq 0. \end{array}$$

On the other hand, for *y* ∈ [*b* ^*∗*^, *x*], by the convexity of *h* on (*b* ^*∗*^, *∞*), we have

(37) $$\begin{array}{c} V_{b^{*}}^{\prime }\left(y\right)-V_{x}^{\prime }\left(y\right)=1-\frac{h^{\prime }\left(y\right)}{h^{\prime }\left(x\right)}\geq 0. \end{array}$$

These give (ii).

Note that *V*_*b*^*∗*^_(*b* ^*∗*^) = *h*(*b* ^*∗*^)/*h*′(*b* ^*∗*^) ≥ *h*(*b* ^*∗*^)/*h*′(*x*) = *V*_*x*_(*b* ^*∗*^) and that (*V*_*b*^*∗*^_ − *V*_*x*_) is nondecreasing on (*b* ^*∗*^, *∞*) because of (ii). Thus, *V*_*b*^*∗*^_(*x*) ≥ *V*_*x*_(*x*); that is, (iii) holds.

For *x* > *b* ^*∗*^, (Γ − *δ*)*V*_*x*_(*x*−) = lim_*y*↑*x*_(Γ − *δ*)*V*_*x*_(*y*) = 0. For *x* ≤ *b* ^*∗*^, we have

(38) Γ−δVb∗x =Γ−δVb∗x−Γ−δVxx− =12σ2Vb∗′′x−Vx′′x−+aVb∗′x−Vx′x  +∫−∞∞Vb∗x+y−Vb∗x−Vb∗′xy1y<1     ×πydy  +∫−∞∞Vxx+y−Vxx−Vx′xy1y<1     ×πydy  −δVb∗x−Vxx≡I1+I2+I3−I4.

Lemmas 11(ii) and 12(i) imply that *I*_1_ ≤ 0, and Lemma 12(iii) implies that *I*_4_ ≥ 0. For *I*_2_ + *I*_3_, we have

(39) I2+I3 =∫−∞∞Vb∗−Vxx+y−Vb∗−VxxVb∗′−Vx′−xy1y<1     −Vb∗′−Vx′xy1y<1πydy =∫−∞0Vb∗−Vxx+y−Vb∗−VxxVb∗′−Vx′−xy1y<1     −Vb∗′−Vx′xy1y<1πydy  +∫0∞Vb∗−Vxx+y−Vb∗−VxxVb∗′−Vx′−xy1y<1     −Vb∗′−Vx′xy1y<1πydy ≡J1+J2.

Applying Lemmas 11(ii) and 12(ii) yields *J*_1_ ≤ 0. For *y* > 0, we obtain

(40) Vb∗−Vxx+y=Vb∗−Vxx= x−b∗+hb∗h′b∗−hxh′x,

which, together with Lemma 12(ii), imply that *J*_2_ = 0. These prove (iv).

We now present the proofs of Theorems 7–10.

**Proof of Theorem 7.**

Define the jump measure of *X* by

(41) $$\begin{array}{c} \mu^{X}=\mu^{X}\left(\omega ,dt,dy\right)=\underset{s}{\sum }1_{\left\{\Delta X_{s}\neq 0\right\}}\delta_{\left(s,\Delta X_{s}\right)}\left(dt,dy\right), \end{array}$$

and its compensator by *υ* = *υ*(*dt*, *dy*) = *dt*Π(*dy*). Then, the Lévy decomposition [36, Theorem 42] gives

(42) Xt=σBt+∫0,t×Ry1y<1μX−υ+at+∫0,t×Ry−y1y<1μX≡ Mt+at+∑0≤s≤tΔXs1y≥1,

where *B* = {*B*_*t*_}_*t*≥0_ is a standard Brownian motion and *M*_*t*_ is a martingale with *M*_0_ = 0.

Note that *ν* is smooth enough for an application of the appropriate version of Itô's formula and the change of variables formula. In fact, if *X* is of bounded variation, then *ν* ∈ *C* ^1^(0, *∞*) and we are allowed to use the change of variables formula [36, Theorem 31]; if *X* has a Gaussian exponent, then *ν* ∈ *C* ^2^(0, *∞*) and we are allowed to use Itô's formula [36, Theorem 32]; and if *X* has unbounded variation and *σ* = 0, then *ν* is twice continuously differentiable almost everywhere but is not in *C* ^2^(0, *∞*) and we can use Meyer-Itô's formula [36, Theorem 70] and product rule formula. In any cases, for any appropriate localization sequence of stopping times {*t*_*n*_,  *n* ≥ 1}, we get under *P*_*x*_

(43) e−δtn∧τξνUtn∧τξξ−νU0ξ =∫0tn∧τξe−δsdMsξ+∫0tn∧τξe−δsΓ−δνUs−ξds  +∑s≤tn∧τξ1ΔLsξ>0e−δs     ×νUs−ξ+ΔXs−ΔLsξ−νUs−ξ+ΔXs        + ν′Us−ξ+ΔXsΔLsξ  −∫0tn∧τξe−δsν′Us−ξdLsξ,

where

(44) Mtξ=∑s≤t1|ΔXs|>0×νUs−ξ+ΔXs−νUs−ξ1|ΔXs|≤1  −ΔXsν′Us−ξ1|ΔXs|≤1−∫0t∫−∞∞νUs−ξ−y−νUs−ξ+yν′Us−ξ1y≤1    ×πydy ds+∫0tν′Us−ξdMs

is a local martingale. The concavity of *ν* implies that *ν*(*x*) − *ν*(*y*) + (*x* − *y*)*ν*′(*y*) ≤ 0 for any *x* ≤ *y*. Taking expectations on both sides of (43) and using conditions (i)–(iii), we obtain

(45) $$\begin{array}{c} E_{x}\left(e^{-\delta \left(t_{n}\wedge \tau^{\xi }\right)}\nu \left(U_{t_{n}\wedge \tau^{\xi }}^{\xi }\right)\right)-\nu \left(x\right)\leq -E_{x}\overset{t_{n}\wedge \tau^{\xi }}{\underset{0}{\int }}e^{-\delta s}dL_{s}^{\xi }. \end{array}$$

Then, letting *n* → *∞* in (45) and recalling that *ξ* is an arbitrary strategy in Ξ, we get

(46) $$\begin{array}{c} \nu \left(x\right)\geq \underset{\xi \in \Xi }{sup}V_{\xi }\left(x\right)=V_{*}\left(x\right). \end{array}$$

This ends the proof of Theorem 7.

**Proof of Theorem 8.**

It follows from (13) and conditions (i) and (ii) that (Γ − *δ*)*V*_*b*_(*x*) ≤ 0 for *x* ∈ (0, *∞*)∖{*b*} and *V*_*b*_′(*x*) ≥ 1 for *x* > 0. Similar to (43), one can show that

(47) e−δtVbUtξ−VbU0ξ =∫0te−δtdNsξ+∫0te−δsΓ−δVbUs−ξds  +∑s≤t1ΔLsξ>0e−δs     ×VbUs−ξ+ΔXs−ΔLsξ−VbUs−ξ+ΔXs  −∫0,te−δsVb′Us−ξdLsξ,c,

where *L*_*s*_^*ξ*,*c*^ is the continuous part of *L*_*s*_^*ξ*^, and

(48) Ntξ=∑s≤t1ΔXs>0  ×VbUs−ξ+ΔXs−VbUs−ξUs−ξ1|ΔXs|≤1    −ΔXsVb′Us−ξ1|ΔXs|≤1−∫0t∫−∞∞VbUs−ξ−y−VbUs−ξUs−ξ1y≤1      +yVb′Us−ξ1y≤1πydy ds+∫0tVb′Us−ξdMs.

Note that *P*(Δ*L*_*s*_^*ξ*^ > 0, Δ*X*_*s*_ < 0) = 0 and that *U*_*s*−_^*ξ*^ + Δ*X*_*s*_ ≥ *b* on {Δ*L*_*s*_^*ξ*^ > 0, Δ*X*_*s*_ > 0}. Consequently, *V*_*b*_′(*U*_*s*−_^*ξ*^ + Δ*X*_*s*_) = 1, and hence

(49) ∑s≤t1ΔLsξ>0e−δsVbUs−ξ+ΔXs−ΔLsξ−VbUs−ξ+ΔXs  =−∑s≤t1ΔLsξ>0e−δsΔLsξ.

Also, for any appropriate localization sequence of stopping times {*t*_*n*_,  *n* ≥ 1}, we have

(50) Exe−δtn∧τξVbUtn∧τξξ−ExVbU0ξ ≤−Ex∫0,tn∧τξe−δsdLsξ.

Letting *n* → *∞* in (50) yields

(51) $$\begin{array}{c} V_{b}\left(x\right)\geq \underset{\xi \in \Xi }{sup}V_{\xi }\left(x\right)=V_{*}\left(x\right). \end{array}$$

However,

(52) $$\begin{array}{c} V_{b}\left(x\right)\leq \underset{\xi \in \Xi }{sup}V_{\xi }\left(x\right)=V_{*}\left(x\right). \end{array}$$

This ends the proof of Theorem 8.

**Proof of Theorem 9.**

If *π*_−_ is completely monotone, it follows from Theorem 6(i) that *h*′(*x*) is strictly convex on (0, *∞*). Then, *V*_*b*^*∗*^_ is concave on (0, *∞*) because of (13). From Lemmas 11(ii) and (iii) and 12(iv), we see that the conditions in Theorem 7 are satisfied. Thus, *V*_*b*_(*x*) ≥ *V*_*∗*_(*x*). Consequently, *V*_*b*_(*x*) = *V*_*∗*_(*x*) and the proof is complete.

**Proof of Theorem 10.**

If *π*_−_ is log-convex on (0, *∞*), it follows from Theorem 6(ii) that *h*(*x*) is strictly convex on (*b* ^*∗*^, *∞*). Then, applying Lemma 12(iv) gives (Γ − *δ*)*V*_*b*^*∗*^_(*x*) ≤ 0 for all *x* > *b* ^*∗*^. The result follows from Theorem 8.
